# A possible effect of montelukast on neurological aging examined by the use of register data

**DOI:** 10.1007/s11096-020-01160-8

**Published:** 2020-10-09

**Authors:** Bjørn Grinde, Henrik Schirmer, Anne Elise Eggen, Ludwig Aigner, Bo Engdahl

**Affiliations:** 1 Division of Mental and Physical Health, Norwegian Institute of Public Health, Oslo, Norway; 2 Department of Clinical Medicine, UiT The Arctic University of Norway, Tromsø, Norway; 3 Department of Cardiology, University Hospital North Norway, Tromsø, Norway; 4 Department of Community Medicine, UiT The Arctic University of Norway, Tromsø, Norway; 5 Institute of Molecular Regenerative Medicine, Spinal Cord Injury and Tissue Regeneration Center Salzburg, Paracelsus Medical University, Salzburg, Austria

**Keywords:** Anti-inflammatory medication, Dementia, Leukotriene, Montelukast, Neurological decline, Prescription database

## Abstract

*Background* The leukotriene receptor antagonist montelukast has been shown to rejuvenate aged brains in rats; however, data on humans are still scarce. *Objective* To investigate if montelukast may alleviate degenerative neurological changes using a register data. *Setting* Norwegian registry data analyses. *Method* The present observational study was based on data from the Norwegian Prescription Database and the Tromsø Study. The former has information regarding the use of prescription medicine; the latter includes tests for brain function such as subjective memory and finger-tapping. Multivariate linear regression analyses were performed to see how the use of various medications correlated with the test results, correcting for likely confounders. *Main outcome measure* Results on seven different tests considered relevant for neurological health were used as outcome. *Results* Previous use of montelukast correlated with improved scores on cognitive or neurological functioning (F = 2.20, *p* = 0.03 in a multivariate test). A range of other medications were tested with the same algorithm, including drugs acting on the immune system, but none of them correlated with (overall) significantly improved test results. *Conclusion* The present data suggest that montelukast may alleviate degenerative neurological changes associated with human aging.

## Impacts on practice

- The data suggest that montelukast may postpone mental aging.
- The dosage used for asthma may not be optimal for possible effects on the brain.
- The data should help initiate a clinical trial to examine the effect of montelukast on aging.

## Introduction

The term ‘inflammaging’ has been coined for an elevated level of low-grade and sterile inflammation [1], which has been associated with age-related decline [2]. That is, inflammatory processes may be causally involved in the aging process in general, and in chronic neurodegenerative diseases such as dementias in particular [3–6]. In the case of dementias, inflammation could be one of the early pathological triggers [7]. Consequently, anti-inflammatory interventions have been proposed for treatment and/or prevention of age-related diseases [8].

Targets for potential anti-inflammatory drugs are, among others, the pathways of the classical lipid-mediators of inflammation. These are for one, the arachidonic acid-derived prostaglandins, a prominent pathway targeted by Cox1/2 inhibitors; and two, the leukotrienes, targeted by leukotriene receptor antagonists. The leukotriene antagonists were developed to treat asthma.

The Cox1/2 inhibitors are included in the umbrella term *non-steroidal anti-inflammatory drugs* (NSAIDs) In addition to these drugs, cortisone-based steroids could be candidates to address inflammaging, although they have, in addition to their anti-inflammatory effects also immune-suppressive activities. In the context of neurodegenerative diseases, NSAIDs have been associated with a reduced risk of dementia [9, 10]. On the other hand, a systematic review concluded that there was no evidence for clinical benefits from the use of NSAIDs or steroidal drugs in the treatment of patients with dementia [11]. This conclusion, however, does not rule out a preventive effect, as the underlying pathology in age-related diseases presumably starts years before the onset of symptoms.

In our recent work, using the *Norwegian Prescription Database* [NorPD], we identified the leukotriene receptor antagonist montelukast as a candidate to reduce the risk of developing dementias [12]. Indeed, a recent case report supports the idea that montelukast might promote cognitive function in patients with dementias [13]. In line with this idea, montelukast treatment restored learning and memory in aged rats, involving a reduction in neuroinflammation, but also a re-activation of neurogenesis as well as a restoration of the blood–brain-barrier integrity [14].

### Aim of the study

The question of whether a particular medication has a positive (or negative) effect on aging is difficult to resolve; for one, because it is ethically questionable to set up clinical trials that involve the use of prescription medicine on healthy individuals; and two, because it would require following individual users over many years. Thus, at least for initial investigations, analyses of data from relevant databases seem to be the best option. A clinical trial will be more acceptable if there is sufficient indirect evidence suggesting a protective effect.

In the present study, we linked information on drug use from NorPD with data on neurological health from the Tromsø study [15]. The linked dataset allowed for considering the effect of any type of medication on brain performance, thus the effect of montelukast was compared with that of other drugs affecting the immune system, focusing on drugs that are typically prescribed over an extended period and to a reasonable number of individuals.

### Ethics approval

The Norwegian Regional Committees for Medical and Health Research Ethics (REK sør-øst) accepted the Project on 18.02.2016 (case 2016/134) with extensions dated 17.03.2017. The project received concession from the Norwegian Data Inspectorate on 21.03.2017 (reference 17/10487). The research was carried out in accordance with relevant guidelines and regulations. The present study did not use data in a form that was person-identifiable. The Tromsø Study includes relevant informed consent. The data files are only available at designated computers within the Norwegian Institute of Public Health.

## Method

### Study design

This is an observational study where we relied on data from two existing databases: NorPD (www.norpd.no) and the Tromsø Study. The Tromsø Study (www.tromsostudy.com) is a prospective cohort study with primarily Caucasian participants [15]. It includes seven surveys (1974–2016], each contains a variety of data concerning both physical and mental health. Data from the last survey, Tromsø 7 (2015–16), formed the basis for the present analyses. Data from Tromsø 6 (2007–08) were investigated to see if they diverged from the results of Tromsø 7. Only individuals 60 years or older at the time of testing were included.

The 2018 version of the NorPD contains information about all prescribed drugs dispensed at pharmacies to individuals in Norway from January 1, 2004 to December 31, 2017. The NorPD classify medication according to the *Anatomical Therapeutic Chemical* (ATC) system [16]. Some of the relevant drugs, particularly acetylsalicylic acid, paracetamol, and NSAIDs, are sold both as prescriptions and over-the-counter. We expect that people receiving prescriptions have more severe conditions, and are more likely to be long term-users.

The Tromsø Study was linked with data from the NorPD by using the Norwegian ID system. The task was performed by Statistics Norway (https://www.ssb.no/en/) in order to ensure that the resulting file did not contain any person-identifiable information. The linked file was used to investigate how the use of various classes of drugs correlated with score on cognition and neurological tests. Multivariate linear regression analyses were performed for this purpose as detailed below. As the users of a particular medication may have a reduced general health compared to non-users, which suggests potentially lower scores on cognitive tests, we considered it pertinent to adjust for health-related confounders.

### Independent and dependent variables

The ATC system offers five levels of classification, the lowest level being specific, active substances. Higher levels were used in cases where individual substances did not have a sufficient number of users. That is, as independent variables we focused on the following medications (ATC code): montelukast (R03DC03), anti-inflammatory and anti-rheumatic products, non-steroids (M01A), glucocorticoids (H02AB), immunosuppressants (L04), acetylsalicylic acid (B01AC06), coxibs (M01AH), inhalation asthma medicine (R03AK and R03BA), and paracetamol (N01BE01). A range of medications affecting the nervous system were tested using the same algorithm, including subgroups of analgesics (N02), antiepileptics (N03), anti-parkinson drugs (N04), psycholeptics (N05), psykoanaleptics (N06), antibacterial agents (J01), and antiviral agents (J05).

Only individuals that received two or more prescriptions prior to the date of testing were considered users. Relatively few people received only a single prescription, and we were uncertain as to whether these individuals had actually taken the drug. The reference group for each drug was those who had received one or none prescriptions of the drug in question.

The data indicating cognitive and neurological functioning came from seven different tests in the Tromsø Study (Table 1). Four are purely cognitive: Mini-Mental State (MMS) examination [17], Digit-symbol coding (part of the Wechsler adult intelligence scale used to examine psychomotor speed, attention, and mental flexibility [18]), Words (Word-test part 1, immediate free recall of 12 nouns), and Subjective memory (memory as assessed by the subject [19]). The latter test was included in Part 1 of Tromsø 7 and had consequently a higher number of participants than the other tests (Part 2), which were on a later day and for a subgroup of the participants.

**Table 1:** Results of tests for montelukast and their controls

Test	Range	Control		Montelukast	
		Score (SD)	N	Score (SD)	N
Cognitive					
MMS	(0, 30)	27.6 (2.3)	5106	27.6 (2.3)	144
Digit-symbol coding	(0, 79)	38.7 (11.1)	4989	38.9 (9.8)	141
Words	(0, 12)	6.8 (1.9)	5093	6.9 (1.8)	143
Subjective memory^a^	(0, 5)	3.9 (1.1)	8045	3.9 (1.1)	220
Neurological					
SPPB	(0, 12)	11.1 (1.6)	5181	11.2 (1.4)	146
Balance	(0, 80)	4.8 (5.4)	4684	4.4 (4.7)	129
Finger-tapping	(19, 85)	54.5 (8.1)	4962	55.1 (7.5)	142
Control					
Grip strength	(2, 71)	29.0 (10.3)	5172	27.4 (10.6)	145
Covariates					
Age	(60, 84)	68.8 (6.1)	5181	68.9 (6.1)	146
DDD	(0, 20.9)	1.5 (1.7)	5181	2.9 (1.9)	146
Number of diseases	(0, 5)	0.5 (0.7)	5181	1.3 (0.8)	146
Self-reported health	(1, 5)	3.7 (0.7)	5181	3.4 (0.7)	146
Sex	Male (%)	2411 (47)	5181	54 (37)	146
Education	High (%)	1968 (38)	5181	57 (39)	146
Smoking	Yes (%)	3321 (64)	5181	91 (62)	146

The scores for controls were the same as for the complete cohort of individuals aged 60 or above. Data on the covariates used in the analyses are given below. *SD* standard deviation, *N* number of participants, *MMS* mini-mental state, *SPPB* short physical performance battery, *DDD d*efined daily dose

^a^This test was performed on a previous day and with a larger number of participants. The covariates data reflect the other tests, but the numbers were not appreciably different in the case of Subjective memory

Three tests were considered relevant for neurological competence: Short Physical Performance Battery (SPPB) [20], Balance (‘Flamingo’ test, the time subject can stand on one foot with eyes closed), and Finger-tapping (tap as many times as possible in 10 s with index finger [21]). These three tests concern coordinated use of muscles where, presumably, the neurological input is vital for the result. In addition, we used measurement of grip strength [22] as a control, considering this test to focus on muscle strength rather than neurology.

All the above variables were treated as continuous and without any particular reference. On all tests, a high score implies improved performance. The number of participants and test scores for montelukast users and their controls (non-montelukast users) are shown in Table 1.

The scores Digit-symbol coding, Finger-tapping and Grip strength were reasonably normal distributed; while MMS, Words, Subjective memory, SPPB and Balance were further from normal distribution. We provided robust estimates of variance, as this strategy is considered to rely less on normal distribution, but may lead to lower precision.

### Confounders

The linked data file allowed for the examination of confounders in the form of: age, sex, education, general health, number of diseases, smoking, and the total number of medicines taken (in the form of *Defined Daily Doses* (DDDs). All these variables, with the exception of age and DDDs, were self-reported. A DDD is the expected average daily maintenance dose for the main indication of a drug [23]. In the present analyses, DDDs refer to the total number of medicines, based on the DDD definition, taken per day averaged over the period from the start of the NorPD in 2004 to the time of attendance. DDDs were used as a proxy for health status. The authors are aware that it is not an ideal proxy, but felt it desirable to include a health parameter that did not rely on self-report. The version of ATC/DDD used was the one available from WHO as of January 1, 2018. The confounders were added in three stages in order to visualize the effect of the adjustments.

*Education* was dichotomized into low (primary/partly secondary education: up to 10 years of schooling or upper secondary education: a minimum of 3 years) and high (Tertiary education). *Smoking* was a category variable with the categories daily smokers, previously daily smoker, and never daily smoker. The former two were merged, thus all declared smokers were tested against non-smokers. *Subjective health* was based on the question “How do you in general consider your own health to be?” with five categories of response. *Number of diseases* was the sum of the following reported diseases: heart attack, heart failure, stroke, asthma, bronchitis, diabetes and cancer. Health and number of diseases were treated as continuous variables.

### Statistical analyses

We conducted analyses with Stata version 15.0. All statistical tests were two-tailed and calculated at a 95% confidence interval (CI). We tested differences in mean test score between users and nonusers of montelukast, or other medications affecting the immune system, for all the seven tests separately by multiple linear regression analysis. All the variables were added simultaneously and thus enforced. We subsequently adjusted for progressively more covariates. In Model 1, we only adjusted for age, sex and, education. In Model 2, DDDs were added as a proxy for general health. In Model 3, we also incorporated self-reported health and number of diseases as continuous variables, as well as smoking as a category variable. Finally, we jointly regressed all seven tests on the above independent variables by multivariate regression “mvreg”, thus testing the joint effect of various medications. Standard errors and significance tests were estimated with the robust estimate in vce (robust).

## Results

### Correlations between drug use and test results

We investigated whether previous use of montelukast, or other drugs that affect the immune system, correlated with test scores related to cognitive and neurological aptitude. Four of the eight tests included focused on cognition, while three reflected neurological control of muscles. A final test, grip strength, was included as a control suggestive of general senescence, but without particular relevance as to brain function. Table 2 lists results for montelukast users and their control group (the data for this control group were identical to the data for the complete cohort at the present level of precision), as well as data on the confounders examined. The number of montelukast users was below 150 for all tests except Subjective memory.

**Table 2:** Effect of montelukast on the various test scores

Test	Model 1^a^		Model 2^b^		Model 3^c^	
	β	95% CI	β	95% CI	β	95% CI
MMS	− 0.10	− 0.46, 0.27	0.04	− 0.34, 0.41	0.10	− 0.28, 0.48
Digit-symbol coding	− 0.07	− 1.50, 1.36	1.47	0.05, 2.89	1.40	− 0.03, 2.83
Words	0.05	− 0.23, 0.34	0.20	− 0.09, 0.49	0.25	− 0.03, 0.54
Subjective memory	− 0.03	− 0.18, 0.12	0.05	− 0.10, 0.21	0.13	− 0.02, 0.28
SPPB	0.09	− 0.12, 0.30	0.34	0.13, 0.55	0.37	0.16, 0.59
Balance	− 0.34	− 1.16, 0.47	0.16	− 0.66, 0.97	0.33	− 0.50, 1.15
Finger-tapping	0.97	− 0.14, 2.08	1.85	0.74, 2.96	1.94	0.79, 3.08
Multivariate test	F = 0.97	*p* = 0.45	F = 1.98	*p* = 0.05	F = 2.20	*p* = 0.03
Grip strength	− 0.15	− 1.21, 0.92	0.52	− 0.53, 1.58	0.62	− 0.44, 1.69

A positive β implies a score above the average of the control group. Differences in β was not statistically significant when 95% CI include both positive and negative limits. The multivariate test examines to what extent the scores of the first seven tests combined are significantly different from the control group. CI confidence interval, MMS mini-mental state, SPPB short physical performance battery

^a^Adjusted for age, sex, and education

^b^Adjusted for age, sex, education, and DDDs

^c^Adjusted for age, sex, education, DDDs, self-reported health, number of diseases, and smoking

Individuals receiving medications are expected to have more health problems, and thus to score lower on both physical and mental tests. We consequently adjusted for relevant confounders. In order to visualize the effect of the confounders, data from three models with progressively more confounders are presented. Table 2 offers the average differences in score compared to controls (β-values), as well as the 95% CI. A positive β implies improved performance. In Model 2 and 3, all the scores were positive, two of them, SPPB and Finger-tapping, significantly so. However, the small number of subjects suggested that a multivariate test, which included scores for all the seven tests, would be more meaningful. This test came out with a *p* = 0.05 in Model 2 and *p* = 0.03 in Model 3. The grip strength of the subjects was not significantly different from that of controls.

In order to put these results in a context, we examined other types of medication affecting the immune system. We limited the analyses to drugs, individual or ATC groups, with a sufficient number of users and an expected long-term use. As to other drugs affecting inflammatory pathways, we investigated ATC code M01A (anti-inflammatory and anti-rheumatic products, non-steroids, here referred to as NSAIDs), and glucocorticoids (ATC code H02AB). Only results based on Model 3 are shown (Table 3). The majority of tests came out with negative β values. The results were generally not significant, with the exception of glucocorticoids having a negative effect on grip strength. Glucocorticoids include all the commonly used steroid type anti-inflammatory agents. NSAIDs had been prescribed to a majority of the cohort, while glucocorticoids were approximately four times as common as montelukast. Similar results were found for coxibs (M01AH), a subgroup of NSAIDs with a different type of action (data not shown).

**Table 3:** Model 3 scores for NSAIDs and glucocorticoids

Test	NSAIDs			Glucocorticoids		
	N	β	95% CI	N	β	95% CI
MMS	3442	0.06	− 0.07, 0.18	590	− 0.01	− 0.20, 0.18
Digit-symbol coding	3368	0.02	− 0.51, 0.54	565	0.13	− 0.63, 0.93
Words	3432	− 0.05	− 0.15, 0.05	584	− 0.10	− 0.26, 0.05
Subjective memory	5342	− 0.03	− 0.08, 0.01	933	0.08	0.00,0.16
SPPB	3495	− 0.05	− 0.13, 0.03	601	− 0.16	0.31, 0.00
Balance	3137	− 0.18	− 0.49, 0.13	492	− 0.23	− 0.62, 0.16
Finger-tapping test	3342	− 0.15	− 0.56, 0.27	568	0.16	− 0.53, 0.84
Multivariate test		F = 1.35	*p* = 0.22		F = 1.30	*p* = 0.25
Grip strength	3488	− 0.19	− 0.54, 0.16	598	− 0.78	− 1.35, − 0.22

A positive β implies a score above the average of the control group. Differences in β was not statistically significant when 95% CI include both positive and negative limits. The multivariate test examines to what extent the scores of the first seven tests combined are significantly different from the control group; that is, non-users of the medication in question. *NSAID* non-steroidal anti-inflammatory drug,* N* number of participants*, CI* confidence interval, *MMS* mini-mental state, *SPPB* short physical performance battery

Drugs affecting other branches of the immune system were included in the form of immunosuppressants (ATC code L04, which includes inhibitors of interleukins and tumour necrosis factors) and acetylsalicylic acid (ATC code B01AC06). Their effects are shown in Table 4. In the case of the immunosuppressants, most of the results were positive, however, the only significantly positive effect was on finger-tapping, while there was a distinct, negative effect on grip strength. As to acetylsalicylic acid, most of the results were negative, except for a significant positive effect on grip strength. In neither case was the multivariate test significant. It should be pointed out that this test considers deviations from mean values, and is thus less meaningful in the case of mixed positive and negative effects.

**Table 4:** Model 3 scores for immunosuppressants and acetylsalicylic acid

Test	Immunosuppressants			Acetylsalicylic acid		
	N	β	95% CI	N	β	95% CI
MMS	172	0.21	− 0.10, 0.52	1223	− 015	− 0.32, 0.02
Digit-symbol coding	163	0.48	− 1.02, 1.99	1176	− 0.61	− 1.28, 0.07
Words	170	0.11	− 0.16, 0.37	1217	− 0.06	− 0.19, 0.07
Subjective memory	265	0.04	− 0.11, 0.18	1924	− 0.06	− 0.13, 0.01
SPPB	176	− 0.12	− 0.45, 0.21	1244	0.01	− 0.11, 0.14
Balance	144	0.16	− 0.56, 0.87	1031	0.26	− 0.12, 0.63
Finger-tapping test	164	1.27	0.18, 2.36	1174	− 0.42	− 0.96,0.12
Multivariate test		F = 1.38	*p* = 0.21		F = 1.43	*p* = 0.19
Grip strength	173	− 2.0	− 3.00, − 0.90	1243	0.72	0.26, 1.18

A positive β implies a score above the average of the control group. Differences in β was not statistically significant when 95% CI include both positive and negative limits. The multivariate test examines to what extent the scores of the first seven tests combined are significantly different from the control group; that is, non-users of the medication in question. *N* number of participants*, CI* confidence interval, *MMS* mini-mental state, *SPPB* short physical performance battery

Other types of drugs were tested with the same algorithm (Model 3), including drugs affecting the nervous system (N02–N06), antibacterial agents (J01), and antiviral agents (J05). They all came out with primarily negative effects (data not shown).

## Discussion

We compared the effect of the leukotriene receptor antagonist montelukast with that of other immune-related drugs on cognitive and neurological fitness. The analyses were based on prescription data from NorPD linked with test results from the Tromsø Study. The results suggest that montelukast might counteract age-related decline in the brain. The suggestion is in line with that of a previous study using data from NorPD alone [12]. In the present study, we had actual test results for brain function, rather than indirect proxies, but while the former study included close to 24,000 individuals on montelukast, N in the present study was generally below 150.

Compared to the beneficial effect of montelukast on cognitive improvement observed in aged rats [14], the apparent effect size in the population studies may seem small. There are several possible explanations as to why humans do not respond better, one prominent factor may be the capacity of montelukast to cross the human blood brain barrier. Although the medication has been shown to penetrate into the brain of both rats and humans, entrance may be a limiting factor [14, 24]. In an uncontrolled case study of individuals suffering from memory loss or mild dementia, daily treatment with 4 × 20 mg of montelukast was reported to improve the condition [13]. It is worth noting that this dose is eight times higher than the dose prescribed to asthma patients in Norway. Ideally, one would hope for a clinical trial where healthy as well as cognitively impaired elderly subjects are given an elevated dose.

In the present work, the best effect of montelukast was on the finger-tapping test (Table 2). This test may be particularly relevant as to neuropsychological functioning. In an examination of stroke patients, this test was the one that best discriminated between survivors with intact cognitive function and those with cognitive decline [25].

The primary indication for the use of montelukast is as additional treatment of asthma in cases where inhalation medication is insufficient. Asthma patients have an increased risk of dementia [26, 27], which is why it seemed pertinent to control for various health variables. It is, however, difficult to know to what extent relevant confounders were included. With all the drugs tested, the results improved when performing the additional adjustments of Model 2 and 3, but only in the case of montelukast did we find a consistent and significant positive effect. The neurological tests seemed to be more positive than the cognitive, which may reflect that the former are more accurate as to probing brain function, and/or are less likely to be distorted by confounders.

The perhaps most interesting part of the results was the distinct difference between montelukast (Table 2) and the other anti-inflammatory agents tested (Tables 3 and 4). It is worth noting that in the case of NSAIDs, previous population based studies suggest that this type of medication may protect against Alzheimer’s disease and other forms of dementia [9, 10]. In the present study, individuals with a history of NSAID prescriptions had reduced score on four of the seven tests. The positive scores regarding montelukast should be seen in relation to this observation.

In order to probe the effect of montelukast, we compared montelukast users to individuals who only use inhalation type asthma medicine (R03AK or R03BA). Montelukast users scored better in all tests in Model 2 and 3 (data not shown), but in this case the multivariate test did not come out as significant (*p* = 0.08), possibly due to the much lower number of controls (generally < 600). We also looked at results based on Tromsø 6. In this study, there were 2587 qualifying participants. The trend, as to a positive effect of montelukast, was the same as in Tromsø 7, but the effects were not significant (data not shown).

The most positive results, next to montelukast, were obtained for immunosuppressants (L04) (Table 4). This is of interest as the group includes sirolimus (L04AA10, better known as rapamycin), which has received considerable attention as an anti-aging drug [28]. Unfortunately, the number of individuals receiving this drug was too small to allow for a separate test.

The medication that yielded the worst scores was paracetamol (N01BE01, data not shown). Paracetamol is a preferred drug for pain management as it may have less side effects than NSAIDs. In Model 2, all the scores were negative and the multivariate test highly significant (*p* = 0.005). One should note that 40% of the sale of this medication in Norway is over the counter (https://www.fhi.no/hn/legemiddelbruk/fakta-om-paracetamol/), those who receive the drug as a prescription may have more serious health problems. The low score of paracetamol users may also reflect that cognitive decline weakens cortical suppression of pain stimuli, which may lead to higher paracetamol use [29]. A similar argument is relevant in the case of NSAIDs, in that some of the more commonly used NSAIDs, such as naproxen and diclofenac, are also sold over the counter. In this case, however, it is assumed that a substantial majority of the medicinal use is based on prescriptions; as this is a cheaper solution for long-term users.

Glucocorticoids had a slightly negative effect on neurological functioning, four out of seven results were negative, but none of them significantly so (Table 3). The negative effect on grip strength was, however, significant. This result is in line with previous observations that glucocorticoids are known to induce myopathy as a common side-effect [30].

### Limitations

The obvious caveat regarding the present study is that the data are based on correlations rather than controlled experiments. Moreover, the observed effect is relatively small and thus vulnerable to confounders we were unable to control for, such as to what extent the conditions associated with prescriptions correlate with brain function.

The low number of participants did not allow for stratifying on how many prescriptions were received or the time between drug use and testing. Information on the use of medicine prior to 2004 was not available; neither was over-the-counter drugs and medications given to patients in nursing homes or hospitals.

## Conclusion

The present analyses add evidence to the notion that montelukast can inhibit neurological and mental decline in humans. The medicine is considered relatively safe, and is therefore of interest for further investigation regarding healthy aging.

## Data Availability

Data were obtained from the NorPD (www.norpd.no) and the Tromsø Study (www.tromsostudy.com). The Norwegian Ethical Committee does not allow us to make the linked data available, but anyone can approach these institutions in order to obtain, at a cost, the relevant data files and apply for the linkage.
